# Tofacitinib Ameliorates Lupus Through Suppression of T Cell Activation Mediated by TGF-Beta Type I Receptor

**DOI:** 10.3389/fimmu.2021.675542

**Published:** 2021-07-29

**Authors:** Qing Yan, Weiwei Chen, Hua Song, Xianming Long, Zhuoya Zhang, Xiaojun Tang, Hongwei Chen, He Lin, Lingyun Sun

**Affiliations:** ^1^Department of Rheumatology and Immunology, Drum Tower Clinical Medical College of Nanjing Medical University, Nanjing, China; ^2^Department of Rheumatology and Immunology, Fujian Provincial Hospital, Shengli Clinical Medical College of Fujian Medical University, Fuzhou, China; ^3^Department of Rheumatology and Immunology, The Affiliated Drum Tower Hospital of Nanjing University Medical School, Nanjing, China

**Keywords:** tofacitinib, transforming growth factor-beta type I receptor, T cell activation, systemic lupus erythematosus, JAK

## Abstract

Autoreactive T cells play a crucial role in the pathogenesis of systemic lupus erythematosus (SLE). TGF-β type I receptor (TGFβRI) is pivotal in determining T cell activation. Here, we showed that TGFβRI expression in naïve CD4^+^ T cells was decreased in SLE patients, especially in those with high disease activity. Moreover, IL-6 was found to downregulate TGFβRI expression through JAK/STAT3 pathway in SLE patients. *In vitro*, the JAK inhibitor tofacitinib inhibited SLE T cell activating by upregulating TGFβRI expression in a dose-dependent manner. In MRL/lpr mice, tofacitinib treatment ameliorated the clinical indicators and lupus nephritis, as evidenced by reduced plasma anti-dsDNA antibody levels, decreased proteinuria, and lower renal histopathological score. Consistently, tofacitinib enhanced TGFβRI expression and inhibited T cell activation *in vivo*. TGFβRI inhibitor SB431542 reversed the effects of tofacitinib on T cell activation. Thus, our results have indicated that tofacitinib can suppress T cell activation by upregulating TGFβRI expression, which provides a possible molecular mechanism underlying clinical efficacy of tofacitinib in treating SLE patients.

## Introduction

Systemic lupus erythematosus (SLE) is a multifactorial autoimmune disease and characterized by excessive activation of T and B cells, as well as the production of various autoantibodies, which affects many organs, especially the kidneys (1, 2). Abnormally activated T cells enhance the autoimmune response in the case of impaired immune tolerance (3). In lupus patients, T cells produce proinflammatory cytokines with aberrant cell signaling properties (4). As conventional immunosuppressive agents, cyclosporine A and tacrolimus have good therapeutic effects by suppressing T cell activation in lupus patients. However, their obvious side effects such as nephrotoxicity limit the clinical use (5). In the last decade, many biological agents that aim to control T cell activation with fewer side effects have been developed for clinical trials, but some of them have failed in the end (5, 6). Thus, there is an urgent need to identify potential targets and develop novel therapeutic strategies against SLE (7).

During the development of autoimmune diseases, the Janus kinase family (JAKs) plays an important role in cytokine signaling, which indicates that targeting JAKs has therapeutic potential in systemic autoimmune diseases (8). Tofacitinib is a pan-JAK inhibitor that preferentially inhibits JAK1 and JAK3 (9), which has shown the efficacy and safety in the treatment of rheumatoid arthritis (10), psoriatic arthritis (11), or ulcerative colitis (12) in many clinical trials. Since the signaling pathways such as the type-I interferon (IFN) signaling involved in the lupus pathogenesis requires JAK/STAT, tofacitinib may also be effective in the treatment of SLE. However, few studies have focused on the therapeutic effects and immunomodulatory mechanisms of tofacitinib in treating SLE.

Transforming growth factor-β1 (TGF-β1) is important for maintaining immune homeostasis and inhibiting the proliferation and differentiation of lymphocytes, especially CD4^+^ T cells. TGF-β1 exerts its biological effects through TGF-β type I receptor (TGFβRI)/TGFβRII/Smad signaling pathway. Our previous studies have demonstrated that TGFβRI acted as a pivotal role in determining T cell activation. When T cell receptor (TCR) was strongly stimulated, the expression of TGFβRI in T cells decreased while TGF-β signaling pathway was inhibited, which led to T cell activation. Our previous studies also found that TGFβRI expression was reduced in the naïve CD4^+^ T cells of untreated and newly diagnosed SLE patients, which suggested that the abnormal expression of TGFβRI might participate in SLE pathogenesis (13).

In this study, we found that TGFβRI expression exhibited an inverse correlation with disease activity in SLE patients and that IL-6 triggered JAK/STAT3 pathway was involved in the downregulation of TGFβRI. Importantly, we found that tofacitinib treatment upregulated the expression of TGFβRI and suppressed the activation of CD4^+^ T cells both *in vitro* and* in vivo*. Moreover, tofacitinib significantly ameliorated lupus nephritis (LN) in MRL/lpr mice. Together, our results identified a novel potential molecular target of tofacitinib for SLE treatment.

## Methods

### Study Population

In total, 77 SLE patients and 41 healthy volunteers from the Department of Rheumatology and Immunology of the Affiliated Drum Tower Hospital of Nanjing University Medical School from October 2018 to August 2020 were enrolled. All SLE patients were diagnosed based on the American College of Rheumatology criteria (revised in 1982). The detailed exclusion criteria were fully described in our previous study (14). The study was approved by the Ethics Committee of our institute, and written informed consent for the collection of venous blood samples was obtained from all subjects.

### Mice and Treatments

Female MRL/lpr mice and C57BL/6 mice (6-week-old) were obtained from Shanghai SLAC Laboratory Animal Co., Ltd. (Shanghai, China) and maintained under specific pathogen-free conditions. The MRL/lpr mice were randomly divided into three groups and treated with vehicle (0.5% methylcellulose/0.025% Tween 20 in ddH2O), tofacitinib (CP-690550; Abmole Bioscience, Houston, TX, 15mg/kg/d), or tofacitinib combined with TGFβRI inhibitor SB431542 (ApexBio, Houston, TX, USA, 10mg/kg). Vehicle and tofacitinib were administered daily by oral gavage for 10 weeks starting at the 10th week, and SB431542 were injected intraperitoneally every day for 2 weeks starting at the 18th week.

### Cell Isolation and T Cell Stimulation

Peripheral blood mononuclear cells (PBMCs) were isolated from the blood samples of SLE patients and healthy controls by Ficoll density gradient centrifugation (STEMCELL Technologies, Vancouver, CA). Human CD4^+^ or naïve CD4^+^ T cells were purified from fresh PBMCs by using the EasySep Human CD4^+^ or Naïve CD4^+^ T Cell Isolation kits (STEMCELL Technologies), stimulated with anti-CD3/CD28 beads (Miltenyi Biotec, Bergisch Gladbach, Germany). Then, CD3/CD28 beads-stimulated naïve CD4^+^ T cells were treated with tofacitinib, STAT3 inhibitor (NSC74859), AKT inhibitor (GSK690693), or p38 MAPK inhibitor (SB203580) for 24 h. NSC74859, GSK690693, and SB203580 were purchased from ApexBio (Houston, TX, USA). Tofacitinib was added at the determined concentrations (0.1 µM, 1 µM, and 10 µM) for 24 h. In some experiments, CD4^+^ T cells were stimulated with anti-CD3/CD28 beads in the presence of recombinant cytokines, such as TGF-β (5 ng/ml; PeproTech, Rocky Hill, NJ) and IL-6 (100 ng/ml; PeproTech).

Spleens were isolated, and the erythrocytes in splenocyte suspensions were lysed with red cell lysate. Mouse CD4^+^ T cells were purified from splenocytes by using the EasySep Mouse CD4^+^ T Cell Isolation kit (STEMCELL Technologies) and processed for real-time PCR or Western blotting analysis.

### Quantitative Real-Time PCR

Total RNA was extracted from naïve CD4^+^ T cells or CD4^+^ T cells using TRIzol reagent (Invitrogen, Carlsbad, CA), and cDNA was synthesized using the HiScript II Q RT SuperMix II (Vazyme Biotech, Nanjing, China). For PCR experiments, reactions containing cDNA, gene primers, and SYBR Green Master Mix (High ROX Premixed, Vazyme Biotech) were run on the StepOnePlus real-time PCR Systems (Applied Biosystems, Foster City, CA). The primer sequences used were as follows: hGAPDH: 5′-ATGGGGAAGGTGAAGGTCG-3′ (forward), 5′-GGGGTCATTGATGGCAACAATA-3′ (reverse); hTGFβRI: 5′-TGCTCGACGATGTTCCATTG-3′ (forward), 5′-CTCTCAAGGCTTCACAGCTC-3′ (reverse); hTGFβRII: 5′- AGGGGTCCGGGAAGGC-3′ (forward), 5′- CTGGGCCTCCATTTCCACAT-3′ (reverse); mGAPDH: 5′-AAGGTCATCCCAGAGCTGAA-3′ (forward), 5′-CTGCTTCACCACCTTCTTGA-3′ (reverse); mTGFβRI: 5′-CAGAGGGCACCACCTTAAAA-3′ (forward), 5′-AATGGTCCTGGCAATTGTTC-3′ (reverse). The relative gene quantification was calculated with the 2^-ΔΔCt^ method.

### Flow Cytometry

The purified CD4^+^ T cells were incubated with the following anti-human antibodies: CD4-PerCP-Cy5.5, CD69-FITC, and CD25-APC. Mouse splenocytes were stained with Fixable Viability Dye eFluor™ 780 (eBioscience) and the following anti-mouse antibodies: CD4-FITC, CD69-BV650, and CD25-APC. All antibodies were purchased from eBioscience. Flow cytometric data were analyzed on a FACS Calibur or Fortessa (BD Biosciences).

### ELISA Analysis

Human IL-6 levels in plasma were measured by ELISA kits from FCMACS (Nanjing, China), and mouse IL-6 and TGF-β1 levels in plasma were measured by ELISA kits from Multiscience Biotech Co., Ltd (Hangzhou, China) according to the manufacturer’s instructions. The murine proteinuria levels were measured by a Bradford protein assay kit (KeyGen Biotech, Nanjing, China). Anti-dsDNA-specific antibody levels in mouse plasma were measured using a Mouse anti-dsDNA ELISA Kit (Shibayagi, Japan).

### Histological and Immunofluorescence Analysis

The kidney tissues were embedded in paraffin for hematoxylin-eosin (H&E), periodic acid-Schiff (PAS), and Masson’s trichrome staining. The kidney pathology was evaluated as previously described (15).

The kidney tissues were snap frozen in liquid nitrogen and embedded in optimal cutting temperature compound. The frozen sections were stained with Alexa Fluor 488-conjugated goat anti-mouse IgG (1:100, Abcam, Cambridge, UK). Next, the sections were stained with DAPI (Sigma). Finally, the sections were photographed by confocal laser scanning microscopy using the Olympus FV3000 Microscope. Mean fluorescence intensity was determined using ImageJ software (NIH). The glomerular IgG staining intensities were graded on a scale of 0–4 (0=absent; 1=faint; 2=moderate; 3=intense; and 4=very intense).

### Western Blotting

Total protein samples were extracted in RIPA buffer with a 1% protease/phosphatase inhibitor cocktail (Cell Signaling Technology, Beverly, MA). Protein samples were separated on a 10% gradient gel (BioRad) and transferred to polyvinylidene fluoride membranes (Millipore). The primary antibodies used were as follows: TGFβRI (1:1000, Santa Cruz Biotechnology), p-STAT3, STAT3, and GAPDH (all are diluted to 1 : 2000 and purchased from Cell Signaling Technology). The protein bands were visualized by a Tanon-5200 chemiluminescent imaging system. Analysis was performed with ImageJ Software.

### Statistical Analysis

Quantitative data are shown as the mean ± SEM. Student’s *t*-tests were applied between two groups. One-way ANOVA was used for comparison among more than two groups. The Pearson r test was used for correlation analysis. All experiments were repeated three times. Statistical analysis was performed with GraphPad Prism version 8 software. A *P* value < 0.05 was considered a significant difference.

## Results

### TGFβRI mRNA Level in Naïve CD4^+^ T Cells Negatively Correlates With Disease Activity Score in SLE Patients

**Figure 1:**
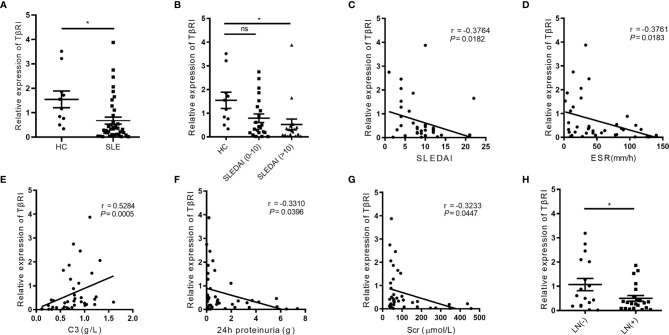
The TGFβRI expression in naïve CD4^+^ T cells is lower in SLE patients and correlates with disease-related variables. **(A)** The level of TGFβRI (TβRI) mRNA in naïve CD4^+^ T cells was compared among SLE patients (n = 39) and healthy controls (HC) (n = 10). **(B)** The relative TGFβRI mRNA level of two lupus subgroups was determined, which included moderate to severe disease activity subgroup (SLE disease activity index, SLEDAI>10) (n = 17) and stable and mild disease activity subgroup (SLEDAI 0-10) (n = 22). **(C–E)** The correlations between TGFβRI mRNA level and SLEDAI, erythrocyte sedimentation rate (ESR), and C3. **(F, G)** The association between TGFβRI mRNA level and 24-h proteinuria and serum creatinine (Scr). **(H)** Lower level of TGFβRI mRNA was found in patients with lupus nephritis (LN) (n = 22) than patients without LN (n = 17). Error bars indicate SEM. *P < 0.05. ns, no significant difference.

We analyzed the TGFβRI mRNA level in naïve CD4^+^ T cells in the SLE patients and healthy controls. The main clinical characteristics of the 39 patients with SLE were summarized in **Table 1**. Significantly reduced TGFβRI expression was found in SLE patients (**Figure 1A**). Based on the SLE disease activity index (SLEDAI) score, SLE patients were divided into two subgroups (0-10, stable and mild disease activity; >10, moderate to severe disease activity). TGFβRI mRNA level in patients with moderate to severe disease activity was lower than that in controls. However, TGFβRI mRNA level in patients with stable and mild disease activity was comparable with controls (**Figure 1B**). We also analyzed the TGFβRII mRNA level, but no significant difference was found between SLE patients and controls (**Supplementary Figure 1A**). Besides, TGFβRII mRNA level in different disease activity subgroups in SLE patients showed no significant difference compared with controls (**Supplementary Figure 1B**).

**Table 1:** Demographic and clinical features of the enrolled SLE patients.

Characteristics	SLE	Healthy control
Age, mean (SD), years	37.41 (14.70)	33.85 (9.10)
Sex, female, n (%)	33 (84.62%)	8 (80%)
Disease duration, mean (SD), months	75.71 (78.70)	
Clinical manifestation, n (%)		
Skin rash	19 (48.72%)	
Arthritis	15 (38.40%)	
Serositis	7 (17.95%)	
Nephritis	22 (56.41%)	
Lung involvement	15 (38.46%)	
Hematologic disorder	24 (61.54%)	
Immunological parameters, n (%)		
ANA positive	39 (100%)	
Anti-Smith positive	19 (48.72%)	
Anti-dsDNA positive	19 (48.72%)	
Low complement C3 or C4	14 (35.90%)	
SLEDAI score, mean (SD)	10.76 (4.77)	

To determine the functional implication of lower TGFβRI expression in SLE patients, we assessed the correlations of the TGFβRI mRNA level with disease-related variables, including SLEDAI score, disease duration, erythrocyte sedimentation rate (ESR), C-reactive protein (CRP), complement C3 and C4, 24-h urine protein, and serum creatinine (Scr). Negative correlations were observed between TGFβRI mRNA level and SLEDAI (**Figure 1C**), ESR (**Figure 1D**), 24-h urinary protein (**Figure 1F**), and Scr (**Figure 1G**). However, C3 level was positively related to TGFβRI mRNA level (**Figure 1E**). Additionally, TGFβRI mRNA level was not associated with disease duration, CRP, and C4 level (**Supplementary Figures 2A–C**). Further analysis showed that SLE patients with LN had lower mRNA expression of TGFβRI than those without LN (**Figure 1H**). In addition, SLE patients with positive anti-dsDNA antibodies had lower mRNA expression of TGFβRI than those without (**Supplementary Figure 2D**). These data indicate that TGFβRI level in naïve CD4^+^ T cells is correlated with disease activities in SLE.

### IL-6 Suppresses TGFβRI Expression Through Activation of JAK/STAT3 Pathway in SLE Patients

**Figure 2:**
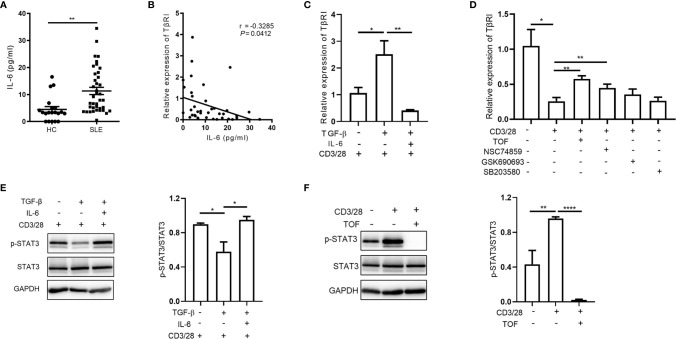
IL-6 downregulates TGFβRI expression *via* JAK/STAT3 pathway. **(A)** Plasma IL-6 levels in SLE patients (n = 39) and HC (n = 20) were measured. **(B)** Correlation between IL-6 and TGFβRI (TβRI) mRNA level was evaluated (n = 39). **(C)** The TGFβRI mRNA level in naïve CD4^+^ T cells from SLE PBMCs was assessed after stimulation with anti-CD3/CD28 beads and TGF-β (5 ng/ml) in the absence or presence of IL-6 (100 ng/ml) for 24 h (n = 4). **(D)** The level of TGFβRI mRNA (n = 3) in anti-CD3/CD28-treated naïve CD4^+^ T cells from SLE PBMCs was assessed after treatment with JAK inhibitor (tofacitinib, TOF), STAT3 inhibitor (NSC74859), AKT inhibitor (GSK690693), or p38 MAPK inhibitor (SB203580) (10 µM). **(E)** The phosphorylated STAT3 (p-STAT3) level in CD4^+^ T cells from HC PBMCs was determined by Western blotting after stimulation with anti-CD3/CD28 beads and TGF-β in the absence or presence of IL-6 for 24 h. p-STAT3 expression was normalized to that of STAT3 (n = 3). **(F)** The protein levels of p-STAT3 and STAT3 in anti-CD3/CD28-treated CD4^+^ T cells from HC PBMCs were assessed after treatment with or without TOF (n = 4). Error bars indicate SEM. *P < 0.05, **P < 0.01, ****P < 0.0001.

To examine the effects of cytokines on TGFβRI expression, naïve CD4^+^ T cells were stimulated with anti-CD3/CD28 beads and cotreated with TGF-β and pro-inflammatory cytokines, such as IL-2, IL-6, IFN-γ, and TNF-α. Only IL-6 was found to suppress the TGF-β-induced upregulation of TGFβRI (13). Moreover, we showed that plasma IL-6 protein levels in SLE patients were significantly higher than those in controls (**Figure 2A**) and negatively correlated with the TGFβRI mRNA level in naïve CD4^+^ T cells (**Figure 2B**). Furthermore, the effects of IL-6 on regulating TGFβRI expression were also observed in naïve CD4^+^ T cells from SLE patients *in vitro* (**Figure 2C**). These data suggest a potential role of IL-6 in suppressing the expression of TGFβRI in SLE patients.

To investigate the molecular mechanism, we further analyzed the downstream signaling pathways activated by IL-6, including the JAK/STAT3, PI3K/AKT, and p38 MAPK pathways. Interestingly, we found that the JAK inhibitor tofacitinib and the STAT3 inhibitor NSC74859, but not AKT inhibitor GSK690693 or p38 MAPK inhibitor SB203580, upregulated TGFβRI expression in anti-CD3/CD28-stimulated naïve CD4^+^ T cells (**Figure 2D**). As shown in **Figure 2E**, TGF-β attenuated STAT3 phosphorylation during CD4^+^ T cell activation, while IL-6 reversed the effect of TGF-β. Upon stimulation, CD4^+^ T cells activated the JAK/STAT3 signaling pathway, which was abrogated by the JAK inhibitor tofacitinib (**Figure 2F**). Collectively, our findings suggested that IL-6 triggered JAK/STAT3 pathway was involved in the downregulation of TGFβRI in naïve CD4^+^ T cells.

### The JAK Inhibitor Tofacitinib Inhibits Activation of CD4^+^ T Cells From SLE Patients by Enhancing TGFβRI Expression

**Figure 3:**
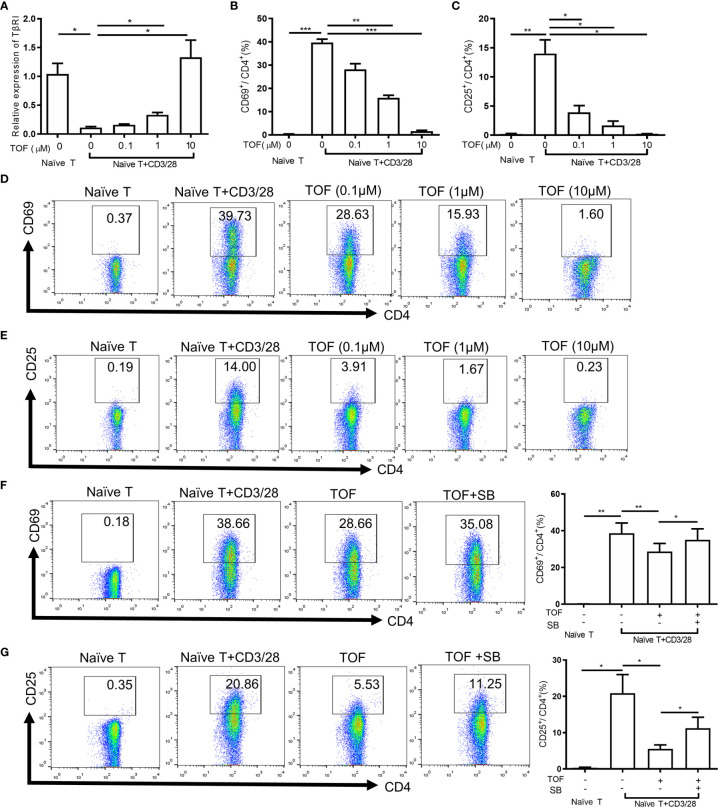
Tofacitinib inhibits the activation of CD4^+^ T cells by increasing TGFβRI expression *in vitro*. Naïve CD4^+^ T cells from PBMCs of SLE patients (n = 4) were stimulated with anti-CD3/CD28 beads in the absence or presence of TOF (0.1-10 µM) for 24 h. **(A)** TGFβRI (TβRI) mRNA level (n = 4) in naïve CD4^+^ cells was analyzed. **(B, D)** The percentages of active CD4^+^ T cells (CD4^+^CD69^+^ T cells) (n = 4) were determined. **(C, E)** The percentages of active CD4^+^ T cells (CD4^+^CD25^+^ T cells) (n = 4) were determined. Purified naïve CD4^+^ T cells from SLE patients (n = 5) were stimulated with anti-CD3/CD28 beads in the absence or presence of TOF (0.1µM) with or without 10µM TGFβRI inhibitor SB431542. **(F)** The percentages of active CD4^+^ T cells (CD4^+^CD69^+^ T cells) (n = 5) were determined. **(G)** The percentages of active CD4^+^ T cells (CD4^+^CD25^+^ T cells) (n = 5) were determined. Error bars indicate SEM. *P < 0.05, **P < 0.01, ***P < 0.001. TOF, tofacitinib; SB, SB431542.

To examine the effect of tofacitinib on the expression of TGFβRI during CD4^+^ T cell activation, naïve CD4^+^ T cells from patients with active SLE were treated with different concentrations of tofacitinib (0.1-10 µM) and anti-CD3/CD28 beads in culture. The decrease of TGFβRI expression in purified naïve CD4^+^ T cells induced by anti-CD3/CD28 beads was reversed in a dose-dependent manner by tofacitinib (**Figure 3A**). However, TGFβRII mRNA expression showed an increasing trend after tofacitinib intervention but did not reach statistical difference (**Supplementary Figure 3A**). Besides, anti-CD3/CD28 beads showed no significant effects on the expression of TGFβRI in PBMCs, and there was no change in TGFβRI mRNA level in the presence of tofacitinib (**Supplementary Figure 3B**). Meanwhile, we stained the activated CD4^+^ T cells with CD4 and the activation markers CD25 and CD69 antibodies and examined them after treatment with tofacitinib by flow cytometry. Tofacitinib markedly reduced the percentages of CD4^+^CD69^+^ T and CD4^+^CD25^+^ T cells dose-dependently (**Figures 3B–E**).

To verify the role of TGFβRI in the inhibition of T cell activation by tofacitinib, the ability of TGFβRI inhibitor SB431542 to block the effect of tofacitinib was studied. SB431542 is a small molecule that suppresses TGF-β pathway by inhibiting TGFβRI activity (16). Purified naïve CD4^+^ T cells from SLE patients were stimulated with anti-CD3/CD28 beads and tofacitinib (0.1 µM) with or without SB431542 (10 µM). Compared with the tofacitinib group, the proportion of activated CD4^+^ T cells increased significantly in the tofacitinib plus SB431542 group (**Figures 3F, G**), suggesting that TGFβRI blockade can reverse the effects of tofacitinib on T cell activation. Taken together, these results suggest that tofacitinib could enhance TGFβRI expression to inhibit T cell activation.

### Tofacitinib Treatment Ameliorates the Clinical Symptoms and Indicators in MRL/lpr Mice

**Figure 4:**
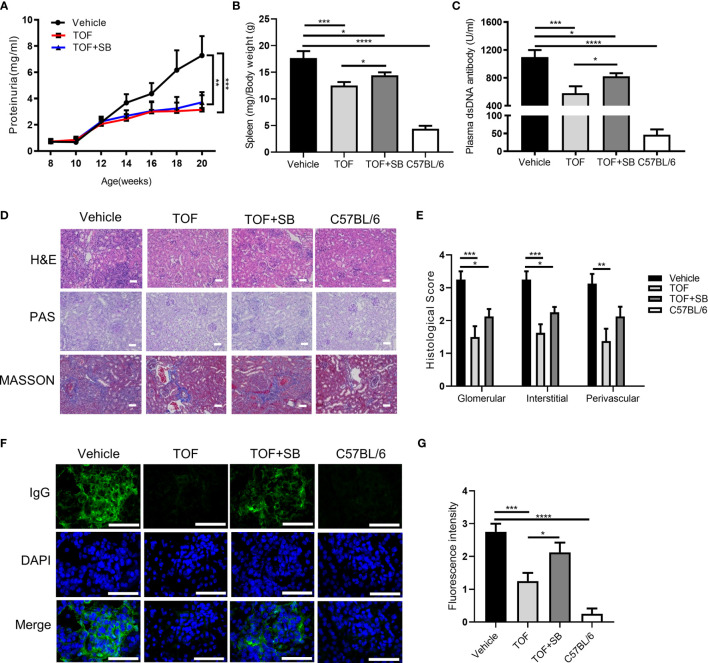
Tofacitinib ameliorates the clinical symptoms and lupus nephritis in MRL/lpr mice. MRL/lpr mice were administered vehicle, tofacitinib alone or combined with SB431542. C57BL/6 mice were the control mice. **(A)** The levels of proteinuria were measured every two weeks. The spleen/body weight ratio **(B)** and plasma anti-dsDNA antibody level **(C)** were determined at the end of the study. **(D)** Representative photomicrograph of renal histology stained with hematoxylin and eosin (H&E), periodic acid-Schiff (PAS), and Masson. Scale bar, 30 µm. **(E)** Histological scores for glomerular, interstitial, and perivascular lesions, according to the above three stains. The control mice did not present any histopathological alteration and the histological score was zero. **(F, G)** Representative images of IgG deposition and fluorescence intensity assessment. Scale bar, 50 µm. n=8 for each group. Error bars indicate SEM. *P<0.05, **P < 0.01, ***P < 0.001 and ****P < 0.0001. TOF, tofacitinib; SB, SB431542.

To determine the efficacy of tofacitinib in treating SLE, we used the widely accepted animal lupus model MRL/lpr mice. MRL/lpr mice developed splenomegaly and various serum autoantibodies compared with mice in the control group (**Figures 4B, C**). As shown in **Figure 4A**, the onset of severe proteinuria was delayed in the tofacitinib-treated group. Proteinuria was significantly reduced in the tofacitinib-treated group at 20 weeks of age (**Figure 4A**). Significant decreases in spleen/body weight ratio were observed in MRL/lpr mice after tofacitinib treatment (**Figure 4B**). Additionally, tofacitinib treatment reduced plasma anti-dsDNA antibody levels compared with those of vehicle treatment (**Figure 4C**).

Since SB431542 could abrogate the suppressive effects of tofacitinib *in vitro*, we investigated whether this effect can be recapitulated *in vivo*. Compared to the tofacitinib treatment group, the spleen/body weight ratio and plasma anti-dsDNA antibody levels were elevated in the tofacitinib plus SB431542 treatment group (**Figures 4B, C**). These results indicated that SB431542 could weaken the therapeutic effects of tofacitinib in MRL/lpr mice.

### Tofacitinib Alleviates LN in MRL/lpr Mice

We evaluated the therapeutic effect of tofacitinib on LN in MRL/lpr mice. Kidney pathology was improved by tofacitinib treatment, as shown by significant reductions in mesangial cell proliferation in glomeruli as detected by PAS staining (**Figures 4D, E**). Additionally, the renal fibrosis expression levels were reduced in the tofacitinib-treated group, as detected by Masson’s trichrome staining (**Figures 4D, E**). After tofacitinib treatment, immunofluorescence staining showed reduced IgG deposition in glomeruli (**Figures 4F, G**). Notably, SB431542 co-treatment reversed these changes (**Figures 4F, G**). Thus, these results from kidney histopathological assessment confirmed that tofacitinib alleviated LN in MRL/lpr mice.

Moreover, we detected the TGFβRI expression in renal tissue by real-time PCR and Western blotting analysis. In contrast to naïve CD4^+^ T cells, renal tissues of MRL/lpr mice showed higher expression of TGFβRI compared to normal controls. After tofacitinib intervention, the expression of TGFβRI was reduced in kidney tissue of MRL/lpr mice (**Supplementary Figure 4**). Therefore, we speculated that tofacitinib treatment might ameliorate renal fibrosis by repressing TGF-β signaling in the kidney.

### Tofacitinib Suppresses CD4^+^ T Cell Activation by Upregulating TGFβRI Expression in MRL/lpr Mice

**Figure 5:**
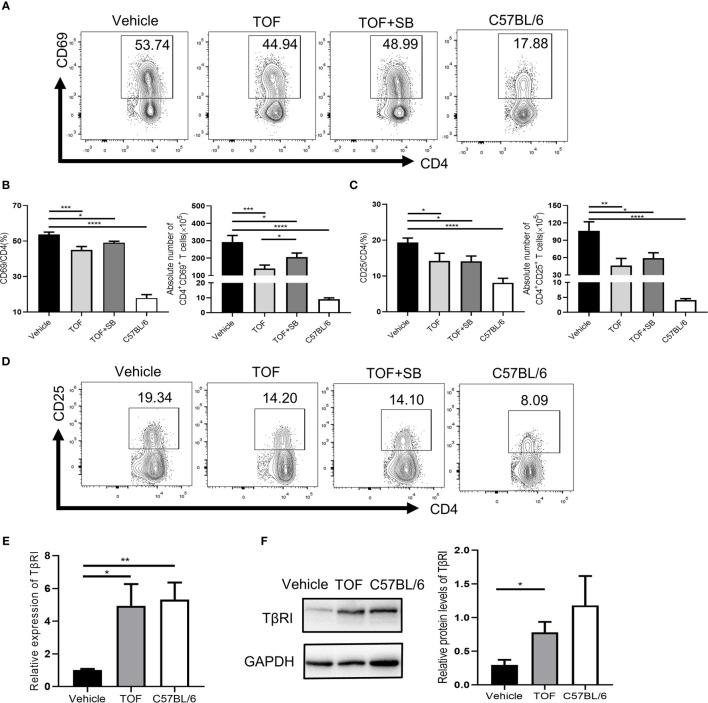
Tofacitinib increases TGFβRI expression and suppresses CD4^+^ T cell activation *in vivo*. **(A)** Percentages of CD4^+^CD69^+^ T cells were determined by flow cytometric analysis. **(B)** The percentages and numbers of CD4^+^CD69^+^ T cells were shown. **(D)** Percentages of CD4^+^CD25^+^ T cells were determined by flow cytometric analysis. **(C)** The percentages and numbers of CD4^+^CD25^+^ T cells were shown. **(E)** The expression of TGFβRI (TβRI) in CD4^+^ T cells from MRL/lpr mice was determined by real-time PCR. n = 8 for each group. **(F)** The protein levels of TGFβRI in CD4^+^ T cells from MRL/lpr mice, as determined by Western blotting (n = 3). Error bars indicate SEM. *P < 0.05, **P < 0.01, ***P < 0.001 and ****P < 0.0001. TOF, tofacitinib; SB, SB431542.

To investigate whether TGFβRI is involved in tofacitinib-induced disease remission in MRL/lpr mice, splenocytes were obtained at the end of the study. As shown in **Figures 5A–D**, the populations of both CD4^+^CD69^+^ and CD4^+^CD25^+^ T cells were significantly decreased in tofacitinib-treated mice compared with vehicle-treated mice. The percentage of CD4^+^CD69^+^ T cells reduced by tofacitinib was partly reversed by SB431542. The absolute number of CD4^+^CD69^+^ T cells decreased significantly after tofacitinib treatment, which was reversed by SB431542 (**Figures 5A, B**). Compared to the tofacitinib treatment group, the absolute number of CD4^+^CD25^+^ T cells in tofacitinib plus SB431542 treatment group also showed an upward trend although the difference was insignificant (**Figure 5C**).

Notably, the TGFβRI mRNA level in the vehicle-treated group was lower than that in the normal controls in CD4^+^ T cells (**Figure 5E**). As expected, TGFβRI expression in CD4^+^ T cells was significantly upregulated in MRL/lpr mice at mRNA (**Figure 5E**) and protein levels (**Figure 5F**) after tofacitinib treatment. We further measured plasma levels of some TGFβRI-related cytokines, such as IL-6 and TGF-β1. As shown in **Supplementary Figure 5**, lupus induced an increase in the levels of pro-inflammatory cytokine IL-6 while decreasing the anti-inflammatory cytokine TGF-β1 levels compared with healthy mice. Tofacitinib intervention reduced IL-6 level but had no effect on TGF-β1 level (**Supplementary Figure 5**).

## Discussion

Autoreactive T cells play a major role in driving and maintaining autoimmune diseases, including SLE (2). Abnormal T cell activation may reduce the TCR activation threshold and impair peripheral tolerance in SLE patients (17). Since TGF-β has suppressive effects on T cell activation (18, 19), TGF-β signaling is involved in the occurrence and development of SLE. In the NZB/WF1 murine model of SLE, the reduction of TGF-β production in T cells is likely to cause immune imbalance and autoantibody production, leading to tissue inflammation (20). Several studies showed that TGFβRI heterodimers or the TGF-β signaling pathway may be defective in SLE patients (21, 22). Our study indicated that the expression of TGFβRI but not TGFβRII in naïve CD4^+^ T cells was related to the disease activity and laboratory indicators such as ESR and C3. Therefore, the low level of TGFβRI but not TGFβRII may be an indicator in identifying patients with severe SLE. A study of female patients with SLE showed a close correlation of serum TGF-β1 levels with severity of renal damage (23). Consistently, we also found that the levels of 24-h urinary protein and serum creatinine were inversely associated with TGFβRI expression. The TGFβRI expression in patients with LN was lower than that in patients without LN. Thus, TGFβRI may play a role in the pathogenesis of SLE.

Previous studies have suggested that IL-6 partially inhibits the upregulation of TGFβRI and TGFβRII in TGF-β-induced trabecular meshwork cells (24). In SLE patients, serum IL-6 was elevated and correlated with disease activity (25). IL-6-deficient mice exhibit resistance to lymphocyte-derived DNA-induced lupus, which causes CD4^+^ T cell activation, anti-dsDNA autoantibody titers, proteinuria, and glomerulonephritis (26). Here, we found that IL-6 could inhibit the upregulation of TGFβRI mediated by TGF-β *in vitro*, and the plasma IL-6 levels in SLE patients were significantly elevated but negatively correlated with TGFβRI mRNA level. This finding may indicate that IL-6 was responsible for the low expression of TGFβRI in SLE patients.

To elucidate the molecular mechanisms by which IL-6 inhibits TGF-β signaling, we investigated the downstream cellular signaling pathways that IL-6 may activate, including the PI3K/AKT, p38 MAPK, and JAK/STAT3 pathways. Our study showed that the addition of JAK or STAT3 but not AKT or p38 MAPK inhibitors significantly upregulated the expression of TGFβRI during CD4^+^ T cell activation. Furthermore, we found that IL-6 reduced the expression of TGFβRI by activating the JAK/STAT3 pathway in CD4^+^ T cells. Consistently, previous studies have reported that IL-6-mediated STAT3 activation can modulate TGF-β signaling, providing a link for cross-talk between the JAK/STAT3 and TGF-β/Smad pathways. For example, the JAK/STAT3 signaling pathway inhibits the TGF-β signaling pathway through the direct interplay of Smad3-STAT3 (27). Hyperactivation of STAT3 desensitizes TGF-β signaling through the inhibition of Smad7 (28). The inhibitory effect of IL-6 on the TGF-β signaling pathway was reduced by siRNA-mediated STAT3 gene knockout (24). Thus, IL-6 might downregulate TGFβRI expression through the JAK/STAT3 signaling pathway in SLE patients. Although the importance of IL-6 in the pathogenesis of lupus has been well documented, pharmacological targeting of IL-6 has not been successful. The human IL-6 receptor inhibitor tocilizumab has achieved clinical benefits, but the concurrent development of severe infections and neutropenia limits its clinical application in SLE (29). Therefore, new targeted therapy with fewer side effects needs to be explored.

The JAK/STAT signaling pathway plays a major role in maintaining immune homeostasis. Mutations in STAT3 can cause lymphoproliferative and autoimmune diseases (30). The potential application of JAK inhibitors in the treatment of SLE is based on the following premises: firstly, JAK/STAT signaling may regulate the expression levels of IFN-regulated factor (IRF)-related genes, which were upregulated in CD3^+^ T cells in active SLE patients (31); secondly, tofacitinib treatment decreased the expression of IFN-signaling genes such as type I IFN genes and STAT1 in MRL/lpr mice (32); and thirdly, tofacitinib could impair the survival rate of renal CD69^+^CD103^+^ tissue-resident memory T cells, which were expanded in the kidney tissues of SLE patients or MRL/lpr mice (33). A previous study has reported that tofacitinib could inhibit the proliferation of CD4^+^ T cells in rheumatoid arthritis (RA) patients, which may be the main mechanism by which tofacitinib treats RA (34). Here, we showed that tofacitinib inhibited T cell activation both *in vitro* and *in vivo*, which was abrogated by SB431542 administration, suggesting TGFβRI was involved in the inhibitory effects of tofacitinib on T cell activation. Our results supported the potential therapeutic application of tofacitinib in SLE.

Consistent with those of previous studies (35, 36), our results showed that tofacitinib significantly ameliorated LN in MRL/lpr mice. Tofacitinib-treated MRL/lpr mice exhibited ameliorated renal function, as evidenced by proteinuria and renal histopathological assessments, compared to those of vehicle-treated animals. Tofacitinib treatment significantly reduced plasma anti-dsDNA antibody levels and IgG deposition in the kidneys, which was reversed by SB431542. In addition, we also found that tofacitinib could alleviate renal fibrosis as detected by Masson staining. The possible mechanism is as follows, it has been shown that TGF-β signaling was suppressed in immune cells but activated in target organs and induced local fibrogenesis in SLE (20). Our research demonstrated the TGFβRI expression was increased remarkably in the kidney tissue of MRL/lpr mice, which is consistent with the previous study (37), and inhibited by tofacitinib treatment.

To date, there have been few clinical studies on the potential application of tofacitinib in patients with SLE. A single-center clinical trial of 10 SLE patients showed that tofacitinib could improve skin and joint symptoms (38). Although several animal studies including ours, have shown that tofacitinib has a beneficial effect on LN (35, 36), more clinical data are needed to clarify the efficacy of tofacitinib treatment in LN.

In summary, we find that reduced TGFβRI expression in naïve CD4^+^ T cells negatively correlates with disease activities in SLE patients. Importantly, we show that the JAK inhibitor tofacitinib alleviates lupus through the upregulation of TGFβRI expression to inhibit CD4^+^ T cell activation. Our findings provide preclinical evidence for the potential therapeutic application of tofacitinib in the treatment of SLE.

## Data Availability Statement

The raw data supporting the conclusions of this article will be made available by the authors, without undue reservation.

## Ethics Statement

The studies involving human participants were reviewed and approved by Nanjing Drum Tower Hospital, The Affiliated Hospital of Nanjing University Medical School. The patients/participants provided their written informed consent to participate in this study. The animal study was reviewed and approved by Nanjing Drum Tower Hospital, The Affiliated Hospital of Nanjing University Medical School.

## Author Contributions

QY and WC participated in performing research, analyzing data, and drafting the manuscript. HS, HL, and ZZ participated in study design and acquisition of clinical data. XL, XT, and HC participated in animal experiments and interpretation of data. LS supervised the whole research. All authors contributed to the article and approved the submitted version.

## Funding

This work was supported by the Key Program of National Natural Science Foundation of China (81930043), the Major International (Regional) Joint Research Project of China (81720108020), the National Natural Science Foundation of China (NSFC) (81771677), and the Natural Science Foundation of Fujian Province (2019J01183).

## Conflict of Interest

The authors declare that the research was conducted in the absence of any commercial or financial relationships that could be construed as a potential conflict of interest.

## Publisher’s Note

All claims expressed in this article are solely those of the authors and do not necessarily represent those of their affiliated organizations, or those of the publisher, the editors and the reviewers. Any product that may be evaluated in this article, or claim that may be made by its manufacturer, is not guaranteed or endorsed by the publisher.
