# Supplementary material for: Tofacitinib Ameliorates Lupus Through Suppression of T Cell Activation Mediated by TGF-Beta Type I Receptor
Source: Front Immunol. 2021 Jul 29;12:675542. doi: 10.3389/fimmu.2021.675542 (PMC8358742; doi:10.3389/fimmu.2021.675542)
Supplement: Supplementary file 1 [file DataSheet_1.docx]

Supplementary Materials

# Supplementary Figures


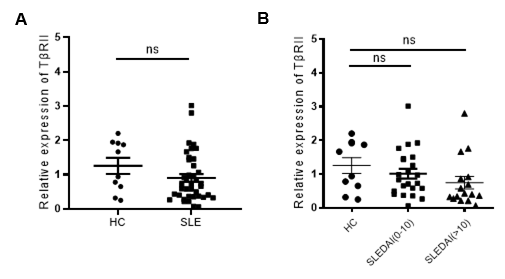


**Supplementary Figure 1.** The level of TGFβRII mRNA in naïve CD4^+^ T cells is compared among SLE patients and healthy controls (HC). (A) There was no significant difference in the TGFβRII (TβRII) expression between SLE patients (n=39) and HC (n=10). (B) No significant difference was found between the moderate to severe disease activity subgroup (SLE disease activity index, SLEDAI>10) (n=17), the stable and mild disease activity subgroup (SLEDAI 0-10) (n=22), and the HC group (n=10). Error bars indicate SEM. ns, no significant difference.


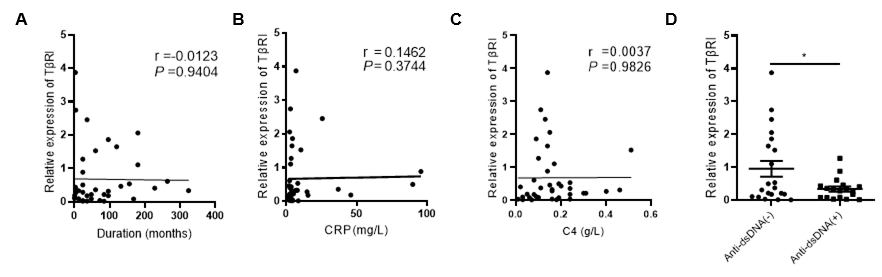


**Supplementary Figure 2.** The TGFβRI expression in naïve CD4^+^ T cells in relation to clinical and laboratory findings from SLE patients. (A-C) The association between TGFβRI (TβRI) mRNA level in naïve CD4^+^ T cells and disease duration (A), C-reactive protein (CRP) (B), and C4 (C). **(**D**)** The relative TGFβRI mRNA level in naïve CD4^+^ T cells was compared between SLE patients with positive anti-dsDNA (n=18) and negative anti-dsDNA antibodies (n=21). Error bars indicate SEM. **P*<0.05.


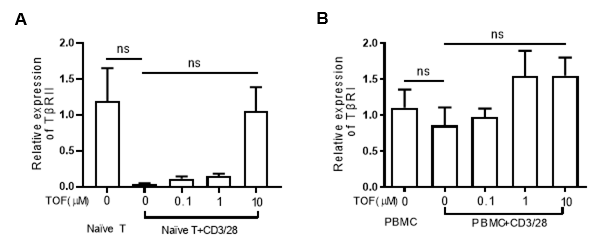


**Supplementary Figure 3.** The influence of tofacitinib on TGFβRI and TGFβRII expression *in vitro*. (A) Naïve CD4^+^ T cells from PBMCs of SLE patients (n=4) were stimulated with anti-CD3/CD28 beads in the absence or presence of TOF (0.1-10 µM) for 24 h. The TGFβRII (TβRII) mRNA level in naïve CD4^+^ cells was analyzed. (B) PBMCs of SLE patients (n=4) were stimulated with anti-CD3/CD28 beads in the absence or presence of TOF (0.1-10 µM) for 24 h. The TGFβRI (TβRI) mRNA level in PBMCs was analyzed. Error bars indicate SEM. ns, no significant difference. TOF: tofacitinib.


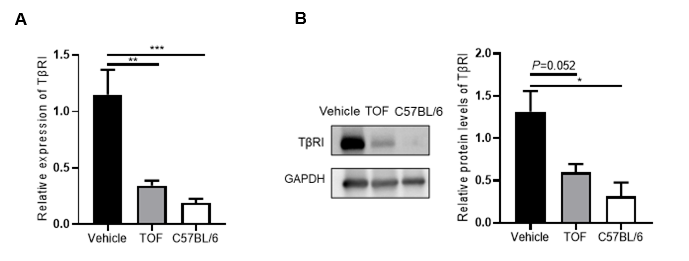


**Supplementary Figure 4.** Tofacitinib inhibits TGFβRI (TβRI) expression in the renal tissues from MRL/lpr mice. (A) The expression of TβRI mRNA in the renal tissues, as determined by real-time PCR, n=5-8 for each group. (B) The protein levels of TβRI in the renal tissues, as determined by Western blotting (n=3). **P*<0.05, ***P*<0.01, ****P*<0.001. TOF: tofacitinib.


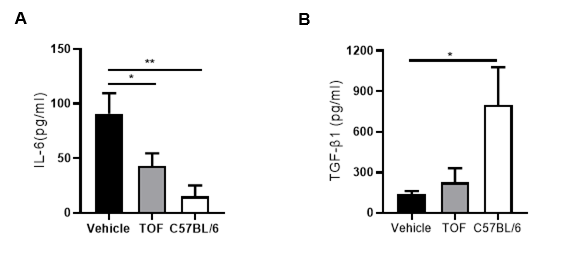


**Supplementary Figure 5.** The influence of tofacitinib on levels of IL-6 and TGF-β1 in plasma of MRL/lpr mice. The levels of IL-6 (A) and TGF-β1 (B) were detected by ELISA, n=5-8 for each group. Error bars indicate SEM. **P*<0.05, ***P*<0.01. TOF: tofacitinib.
